# Improving visualization of 4D flow cardiovascular magnetic resonance with four-dimensional angiographic data: generation of a 4D phase-contrast magnetic resonance CardioAngiography (4D PC-MRCA)

**DOI:** 10.1186/s12968-017-0360-8

**Published:** 2017-06-23

**Authors:** Mariana Bustamante, Vikas Gupta, Carl-Johan Carlhäll, Tino Ebbers

**Affiliations:** 1 Division of Cardiovascular Medicine, Department of Medical and Health Sciences, Linköping University, Linköping, Sweden; 2 Center for Medical Image Science and Visualization (CMIV), Linköping University, Linköping, Sweden; 3 Department of Clinical Physiology, Department of Medical and Health Sciences, Linköping University, Linköping, Sweden

**Keywords:** Computer-Assisted Image Analysis, 4D flow cardiovascular magnetic resonance (4D flow CMR), Phase-Contrast Magnetic Resonance Angiography (PC-MRA)

## Abstract

Magnetic Resonance Angiography (MRA) and Phase-Contrast MRA (PC-MRA) approaches used for assessment of cardiovascular morphology typically result in data containing information from the entire cardiac cycle combined into one 2D or 3D image. Information specific to each timeframe of the cardiac cycle is, however, lost in this process. This study proposes a novel technique, called Phase-Contrast Magnetic Resonance CardioAngiography (4D PC-MRCA), that utilizes the full potential of 4D Flow CMR when generating temporally resolved PC-MRA data to improve visualization of the heart and major vessels throughout the cardiac cycle. Using non-rigid registration between the timeframes of the 4D Flow CMR acquisition, the technique concentrates information from the entire cardiac cycle into an angiographic dataset at one specific timeframe, taking movement over the cardiac cycle into account. Registration between the timeframes is used once more to generate a time-resolved angiography. The method was evaluated in ten healthy volunteers. Visual comparison of the 4D PC-MRCAs versus PC-MRAs generated from 4D Flow CMR using the traditional approach was performed by two observers using Maximum Intensity Projections (MIPs). The 4D PC-MRCAs resulted in better visibility of the main anatomical regions of the cardiovascular system, especially where cardiac or vessel motion was present. The proposed method represents an improvement over previous PC-MRA generation techniques that rely on 4D Flow CMR, as it effectively utilizes all the information available in the acquisition. The 4D PC-MRCA can be used to visualize the motion of the heart and major vessels throughout the entire cardiac cycle.

**Electronic supplementary material:**

The online version of this article (doi:10.1186/s12968-017-0360-8) contains supplementary material, which is available to authorized users.

## Introduction

Magnetic Resonance Angiography (MRA) is a commonly used technique for vessel visualization, utilized routinely to detect or evaluate pathologies such as stenoses, aneurysms or vascular anomalies. Conventional MRA commonly relies on the use of external agents for contrast enhancement, as opposed to Phase-Contrast MRA (PC-MRA) where the contrast is generated using phase differences in the MR signal [1, 2]. PC-MRA data are typically acquired without cardiac gating in a breath-hold. Consequently, any motion present during the cardiac cycle will be averaged in the resulting image, likely becoming difficult to perceive.

Three-dimensional (3D) cine (time-resolved) phase-contrast cardiovascular magnetic resonance (CMR) with three-directional velocity-encoding (4D Flow CMR) is a technique that permits visualization and evaluation of the pulsatile blood flows in the chambers of the heart and great thoracic vessels over the cardiac cycle in a single acquisition [3]. In 4D Flow CMR, data is acquired using cardiac electrocardiography (ECG) gating. Therefore, the resulting images also include information about the motion of the heart and vessels over the cardiac cycle. Considerably similar images to those obtained when using PC-MRA are often generated from the 4D Flow CMR data for orientation and visualization purposes. During this process, motion over the cardiac cycle is typically averaged and consequently lost.

Different methods for 3D PC-MRA data generation from 4D Flow CMR have been proposed and evaluated [4–6]. These methods combine and average the velocity and magnitude information over the cardiac cycle. The velocities yield higher intensities in the resulting image in areas of high blood flow, while the magnitude signal adds morphological information and mitigates noise in areas of very low signal, such as the lungs. For *N* timeframes, [3, 5], and [7], proposed the following equation:

1 $$ 3\mathrm{D}\mathrm{P}\mathrm{C}-\mathrm{M}\mathrm{R}\mathrm{A}=\frac{1}{N}{\displaystyle \sum_{t=1}^N}{M}^2(t)*\sqrt{\left({V}_x^2(t)+{V}_y^2(t)+{V}_z^2(t)\right)} $$

While [6] proposed the following:

2 $$ 3\mathrm{D}\mathrm{P}\mathrm{C}-\mathrm{M}\mathrm{R}\mathrm{A}=\sqrt{\frac{1}{N}{\displaystyle \sum_{t=1}^N}{M}^2(t)*\left({V}_x^2(t)+{V}_y^2(t)+{V}_z^2(t)\right)} $$

**Fig. 1:**
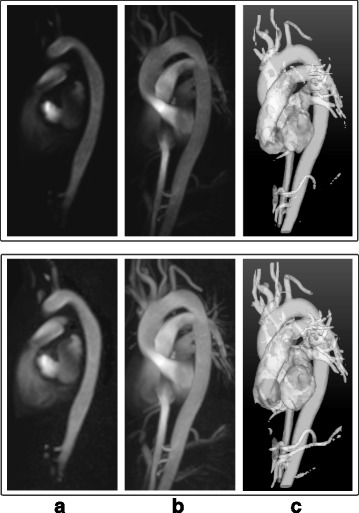
3D PC-MRA visualizations: 3D PC-MRA data created from a 4D Flow CMR dataset using the method presented in [6]. **a** 2D coronal slice of the volume, **b** Maximum intensity projection (MIP), and **c** Isosurface rendering

In these equations, *t* is the corresponding timeframe, *V*_*x*_, *V*_*y*_ and *V*_*z*_ are the blood flow velocity components in three spatial directions, and *M* is the magnitude of the signals acquired during the 4D Flow CMR acquisition. The 3D PC-MRAs generated from 4D Flow CMR data are often visualized using Maximum Intensity Projections (MIPs) or isosurface renderings, resulting in slightly different angiographic images, as shown in Fig. 1.

In addition to vessel visualization, the generated data can also be used to segment the blood lumen, which may be necessary for further analyses, such as flow assessments [8–10], or wall shear stress (WSS) estimation [11]. A 3D PC-MRA generated from 4D Flow CMR using the described approaches will have the same features and limitations as a standard PC-MRA, such as the smoothing of moving structures. This hampers visualization of the heart chambers and can affect the calculation of parameters that rely on segmentation. For example, when estimating WSS from a time-averaged PC-MRA, the movement of the aortic wall due to the natural distension and recoil motion of the aorta over the cardiac cycle is usually not accounted for [12].

The goal of this study is to present and evaluate a novel technique, called 4D Phase-Contrast Magnetic Resonance CardioAngiography (4D PC-MRCA), that utilizes the full potential of 4D Flow CMR for the visualization of heart chambers (“cardio”) and vessels (“angio”).

The purpose of creating a 4D PC-MRCA is two-fold: first, to allow the visualization of both the motion of the heart and major vessels throughout the cardiac cycle by generating an image that is time-resolved; and second, to enhance the intensities in the heart ventricles and atria to improve their discernibility. The 4D PC-MRCA is not intended to substitute the conventional contrast-enhanced MR angiography (CE-MRA); instead, it aims to improve the visualization of 4D Flow CMR.

## Methods

### Creation of a 4D Phase-Contrast Magnetic Resonance CardioAngiography (4D PC-MRCA)

A 4D Flow CMR dataset can be seen as a series of three-dimensional volumes over time (4D), where each volume contains magnitude information as well as three-directional velocity information.

In order to allow for visualization of temporal changes in the geometry of both the thoracic vessels and the heart over a cardiac cycle, the 4D PC-MRCAs were generated using the following steps:

1. PC-MRA data were generated for every available timeframe using equation 3 for each voxel in the magnitude and velocity data included in the 4D Flow CMR dataset.
  3 $$ \mathrm{P}\mathrm{C}-\mathrm{M}\mathrm{R}\mathrm{A}(t)= M(t)*\left({V}_x^2(t)+{V}_y^2(t)+{V}_z^2\right( t{\left)\right)}^{\gamma} $$
  Gamma (*γ*) correction of 0.2 was used to enhance the velocity values and to make sure that the lower velocities, such as those present in the heart chambers, were also included in the image. The resulting 4D image retains the information specific to each timeframe of the cardiac cycle.
2. All the timeframes of the 4D Flow CMR magnitude image were aligned to one timeframe of the cardiac cycle using non-rigid registration. For the selected datasets belonging to healthy volunteers, this timeframe was chosen to be during mid-diastole (diastasis), when the heart was at an intermediate position between early and late ventricular diastole. For *N* timeframes in a cardiac cycle, this resulted in *N* − 1 transformations, *B*_*t*_, each corresponding to one timeframe, *t*. See Fig. 2 for further clarification of this step.

**Fig. 2:**
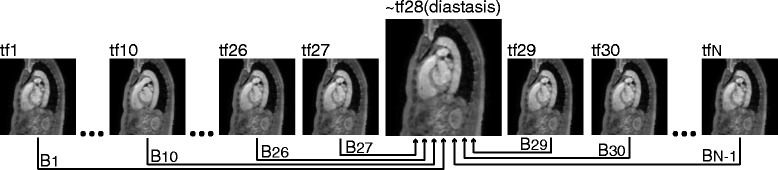
4D PC-MRCA creation, step 2: Registration of every timeframe of the average magnitude image to a timeframe in mid-diastole (diastasis), resulting in *N* − 1 transformations, *B*_*t*_
3. Each timeframe, *t*, of the PC-MRA data created in step 1 was transformed using the corresponding deformation field, *B*_*t*_, to generate a set of images with intensities that depend on the blood flow patterns at each timeframe, but with the shape and morphology expected in the chosen diastasis timeframe.
4. A 3D PC-MRCA was calculated as an MIP of these images over time. These data contain high contrast in all sections of the cardiovascular system where high blood flow occurs at least once during the heartbeat, and morphologically corresponds to one specific timeframe of the cardiac cycle. The maximum of the images over time was preferred over the average in order to preserve visibility in the heart chambers, where the contrast is usually lower.
5. A new set of registrations were executed. In this case, the magnitude image of the chosen diastasis timeframe was registered to the remaining timeframes of the cardiac cycle. This resulted in *N* − 1 transformations, *F*_*t*_, one for each timeframe, *t*, that were then applied to the 3D PC-MRCA in order to obtain a time-resolved (four-dimensional) PC-MRCA.

A non-rigid registration method based on the Morphon algorithm was used for this study [13]. The implementation uses diffeomorphic field accumulation, together with fluid and elastic regularization of the displacement fields in order to generate physically plausible deformations [14]. The regularization parameters were initially determined following the settings from a previous study where the same registration method was used on clinical computed tomography (CT) images [15]. Further tuning of these parameters for the current application resulted in the following settings:

- Number of scales: 2
- Number of iterations per scale: 3
- Gaussian kernel with *σ* of 1.5 pixels for both fluid and elastic regularization.

The 4D PC-MRCA generation tool created for this project was implemented using MATLAB (release 2015b, The MathWorks, Inc., Natick, Massachusetts, USA).

### Study population

Evaluation of the proposed method was performed on ten 4D Flow CMR datasets acquired from healthy volunteers with no prior history of cardiovascular disease or cardiovascular medication. The group’s mean age was 66±4, within the range 59–71, and included 9 females and 1 male.

4D Flow CMR examinations were performed on a clinical 3 T Philips Ingenia scanner (Philips Healthcare, Best, the Netherlands) and were acquired during free-breathing, using a navigator gated gradient-echo pulse sequence with interleaved three-directional flow-encoding and retrospective vector cardiogram controlled cardiac gating. All subjects were injected with a Gd contrast agent (Magnevist, Bayer Schering Pharma AG) prior to the acquisition for a late-enhancement study. Scan parameters included: Candy cane view adjusted to cover both ventricles, velocity encoding (VENC) 120 cm/s, flip angle 10^∘^, echo time 2.6 ms, repetition time 4.4 ms, parallel imaging (SENSE) speed up factor 3 (AP direction), k-space segmentation factor 3, acquired temporal resolution of 52.8 ms, spatial resolution 2.7 × 2.7 × 2.7 *mm* ^3^, and elliptical k-space acquisition. The typical scan time was 7–8 min excluding and 10–15 min including the navigator gating.

The 4D Flow CMR data were corrected for concomitant gradient fields on the CMR scanner. Offline processing corrected for phase wraps using a temporal phase unwrapping method [16], and background phase errors were corrected using a weighted 2nd order polynomial fit to the static tissue [17].

Noise caused by the presence of air in the velocity data was suppressed by thresholding the signal intensity in the magnitude data. Values lower than 10% of the maximum magnitude value were ignored, as proposed in [7]. Also, voxels with an absolute velocity value more than 50% higher than the velocity encoding (VENC) were ignored. This suppressed a few scattered voxels (on average 0.011% of all voxels) with very noisy velocity information but a magnitude value above the magnitude threshold.

### Evaluation

4D PC-MRCA and 3D PC-MRA data were generated for each dataset, and MIPs of each method were used to assess the resulting images. Anatomical regions of the cardiovascular system were scored according to their visibility in the projections, emphasizing the diagnostic quality of the evaluated image. The scoring was performed independently by two observers: a clinician with 5 years of experience in cardiovascular imaging, and an image analyst with 3 years of experience in cardiovascular imaging. Each score was based on the following scale: 1 = poor, region not visible, of no diagnostic quality; 2 = fair, region faintly visible, but not of diagnostic quality; 3 = good, complete region visible, of diagnostic quality; 4 = excellent, region clearly defined, of excellent diagnostic quality. The scores for each method were compared using the Wilcoxon rank-sum test. A *p*-value < 0.05 was considered to represent a significant difference between techniques.

To serve as comparison to the proposed method, 3D PC-MRA data were generated for this study using the method presented by Hennemuth et al. (2) since, similar to the 4D PC-MRCA, this method also retains higher intensities inside the heart. In contrast, other previously proposed 3D PC-MRA techniques focus mostly on the vessels and apply strong background suppression, thereby leaving very low intensities in and around the heart.

## Results

**Fig. 3:**
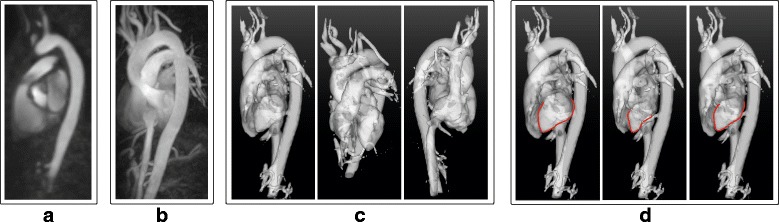
Visualization of a 4D PC-MRCA: **a** Coronal slice at a mid-diastolic timeframe. **b** Maximum Intensity Projection (MIP) at a mid-diastolic timeframe. **c** Isosurface renderings from three different points of view at an end-diastolic timeframe. **d** Isosurface renderings for three timeframes of the cardiac cycle (end-diastole, systole, mid-diastole). For visualization purposes, the left ventricle has been delineated in red for each timeframe

Figure 3 shows different visualization methods applied to a 4D PC-MRCA in parts (a), (b), and (c). Additionally, part (d) shows isosurface renderings for three different timeframes of the four-dimensional image in which the approximate contour of the left ventricle has been indicated in red for visualization purposes. The corresponding movies for the entire cardiac cycle, including all the available timeframes, have been included as supplementary material.

An MIP for each timeframe available in the dataset can be calculated from the 4D PC-MRCA. In contrast, the 3D PC-MRA results in a single volume not representing any particular timeframe. Videos corresponding to each MIP were created using 36 angle projections, and have been included as supplementary material.

**Fig. 4:**
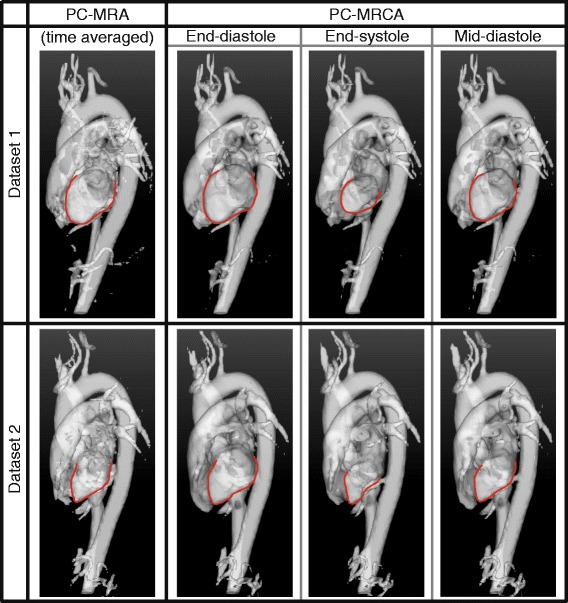
Method comparison using isosurfaces: 3D PC-MRA and 4D PC-MRCA isosurface renderings generated for two 4D Flow CMR datasets (*top and bottom*). The left ventricle has been highlighted in red in all the images for visualization purposes

**Fig. 5:**
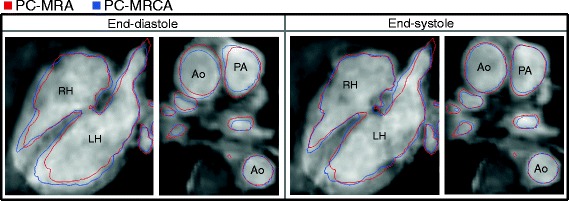
Comparison of isosurface boundaries: PC-MRA (*red*) and PC-MRCA (*blue*) at two timeframes. The visible regions are: Left ventricle and atrium (*left heart*, LH), right ventricle and atrium (*right heart*, RH), aorta (Ao), and pulmonary artery (PA)

Isosurface renderings generated from 3D PC-MRAs and 4D PC-MRCAs for two datasets are shown in Fig. 4, only three different timeframes of the 4D image are shown due to space constraints. The corresponding movies for the entire cardiac cycle have been included as supplementary material. Additionally, the boundaries of the isosurfaces of the PC-MRA and PC-MRCA at two timeframes are compared in Fig. 5. Videos including the entire cardiac cycle have been included as supplementary material. Note that the 3D PC-MRA data is not time-resolved; consequently, the same isosurface was used for the different timeframes pictured. In this image, discrepancies can be observed between the PC-MRA and the location of the vessels as a consequence of vessel motion, especially during end-diastole.

**Fig. 6:**
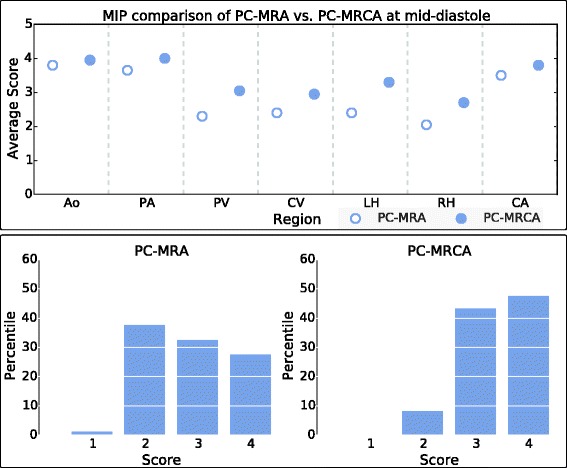
Visual evaluation results: Top: Average scores obtained by the MIPs of the PC-MRA and PC-MRCA at mid-diastole. The regions evaluated were: Aorta (Ao), pulmonary arteries (PA), pulmonary veins (PV), caval veins (CV), left ventricle and atrium (LH), right ventricle and atrium (RH), and carotid arteries (CA). The differences for LH, RH, PV, and CV are statistically significant (*p* < 0.05). Bottom: Percentile of scores obtained for each value in the scale by the MIPs of the PC-MRA (*left*) and the PC-MRCA at mid-diastole (*right*). The difference between the scores obtained by the PC-MRA and those obtained by the PC-MRCA is statistically significant (*p* < 0.001)

Figure 6 (top) shows the average scores from the MIPs of 3D PC-MRA and 4D PC-MRCA data at a mid-diastolic timeframe. Figure 6 (bottom) illustrates the differences between the angiographies according to the percentiles obtained by each score in the scale. Detailed scores can be seen in Table 1. The differences between the scores obtained by both techniques were highly significant (4.97), with p < 0.001.

**Table 1:** Detailed visual evaluation scores

Region	3D PC-MRA				4D PC-MRCA (mid-diastole)			
	Score:1	Score:2	Score:3	Score:4	Score:1	Score:2	Score:3	Score:4
Aorta	[0,0]	[0,0]	[2,2]	[8,8]	[0,0]	[0,0]	[1,0]	[9,10]
Pulmonary arteries	[0,0]	[0,0]	[2,5]	[8,5]	[0,0]	[0,0]	[0,0]	[10,10]
Pulmonary veins	[0,0]	[7,7]	[3,3]	[0,0]	[0,0]	[1,1]	[7,8]	[2,1]
Caval veins	[0,0]	[8,4]	[2,6]	[0,0]	[0,0]	[1,2]	[9,6]	[0,2]
Left ventricle and atrium	[0,0]	[5,7]	[5,3]	[0,0]	[0,0]	[0,1]	[5,7]	[5,2]
Right ventricle and atrium	[2,0]	[8,7]	[0,3]	[0,0]	[0,0]	[3,3]	[7,7]	[0,0]
Carotid arteries	[0,0]	[0,0]	[4,6]	[6,4]	[0,0]	[0,0]	[1,3]	[9,7]

Number of datasets that received the indicated score during visual evaluation of the MIP generated for the PC-MRA and PC-MRCA at mid-diastole. [Observer 1, Observer 2]. Scale: 1 = poor, region not visible, of no diagnostic quality; 2 = fair, region faintly visible, but not of diagnostic quality; 3 = good, complete region visible, of diagnostic quality; 4 = excellent, region clearly defined, of excellent diagnostic quality

The 4D PC-MRCAs received higher scores than the 3D PC-MRAs in most cases. Noteworthy results were the higher scores obtained when evaluating the left and right chambers of the heart.

## Discussion

The proposed method to calculate 4D PC-MRCA data from 4D Flow CMR permits visualization of the cardiovascular system from several viewpoints, together with the possibility of observing the motion of the heart and vessels during the entire cardiac cycle. This represents an improvement over the existing techniques for 3D PC-MRA generation from 4D Flow CMR and can be an advantage in clinical practice, particularly when the type of clinical question being assessed is influenced by cardiovascular wall motion.

During the visual evaluation, 4D PC-MRCA outperformed 3D PC-MRA obtaining higher averages and scores of mainly threes and fours on the defined scale, compared to mostly twos or threes for the PC-MRA (see Fig. 6). Furthermore, the movement of the myocardial and vascular walls over the cardiac cycle produced disparities in the 3D PC-MRA in several timeframes. These problems were especially visible in the ascending aorta and pulmonary artery, two regions frequently assessed with angiographic images. An example of such discrepancies can be seen in Fig. 5 at end-diastole.

Accurate depiction of the vessel wall over the cardiac cycle is important in obtaining accurate measurements of vessel size, as this can vary significantly throughout the cardiac cycle. It also enables an accurate computation of certain parameters, such as WSS, which has been studied intensively in the ascending aorta and carotid arteries. Furthermore, previous studies have emphasized the necessity for accurate vessel wall location for WSS calculation [12, 18]. Its analysis, however, has so far been focused on peak values. Accurate segmentation of the vessel wall over the complete cardiac cycle would allow for computation of additional interesting WSS parameters, such as oscillatory shear index (OSI) [19].

The presented method is completely automatic. Consequently, it can be easily added to the existing post-processing algorithms already required by 4D Flow CMR acquisitions, e.g., transformation from DICOM to other formats, background correction, phase unwrapping, etc.

Although the resulting images are not precise enough to be used directly in the segmentation of the cardiac ventricles and atria, the 4D PC-MRCAs enabled sufficient description of the location, motion, and size of the cardiac chambers to fulfill the goal of visualization of the cardiovascular system over the entire cardiac cycle. It was not the aim of this study to evaluate a potential segmentation method resulting from the PC-MRCA data. However, a combination of the PC-MRCA and advanced segmentation techniques is expected to result in better segmentation of the cardiac chambers on 4D Flow MR images.

We have evaluated the method on 10 healthy volunteers. The presented method is, however, also expected to be able to aid in the visualization of subjects with pathological features such as vascular disease, heart valve disease, and myocardial disease affecting the size or shape of the heart. The PC-MRCA generated from a dataset with a dilated left ventricle with at least moderately depressed systolic function has been included as supplementary material. In pathological cases involving severe jet flow, signal void may occur in the PC-MRI data, which is known to affect traditional PC-MRA. This signal void is also expected to affect the PC-MRCA, but possibly to a lesser extent. The concept used in computing the PC-MRCA allows for the utilization of information from the whole cardiac cycle. Jet flow typically only occurs in either systole or diastole. By utilizing information from the complete cardiac cycle, the cardiac phases without signal void can be used in the PC-MRCA. This could be a subject of future research.

The specific equation used to combine the magnitude and velocity images in step 1 of the described method should depend on the required aim of the PC-MRCA images. In this evaluation, we did not focus on background suppression in favor of increasing the intensities in areas of lower flow velocities. Changes to the proposed equation can be made in order to enhance different areas of the image or to achieve stronger background suppression. For instance, an optimal depiction of the aortic wall could be achieved by replacing equation (3) with an equation similar to equation (1). This might produce a slightly better delineation of the aorta, but at the cost of a lower quality depiction of other anatomical regions. Moreover, using average instead of maximum to create the diastasis-located image in step 4 of the PC-MRCA creation could be particularly useful if the input data is noisy.

The generation of one 4D PC-MRCA took on average twenty minutes on a system with 64GB of RAM and a 6-core 3.5GHz processor. The current implementation included parallel execution of multiple registrations. Further improvements to the running time can be achieved by employing GPU programming, or by taking advantage of the fact that the second set of registrations required (step 5 of the method) correspond to the inverse of the transformations obtained during the first set of registrations (step 2 of the method). Consequently, both sets of transformations, *B*_*t*_ and *F*_*t*_ for a timeframe *t*, could be calculated together in step 2.

As an additional measure of the accuracy of the non-rigid registration, the inverse consistency of the deformation fields *B*_*t*_ and *F*_*t*_ was assessed by comparing the image intensities of each timeframe of the magnitude image after applying both the backward and forward registrations. The magnitude images had intensities in the range [0-1], thus the maximum possible error is 1. The sum of squared errors (± standard deviation) obtained for all the datasets included in the study was 8.940 * 10^− 5^ ± 2.811 * 10^− 4^. This inverse consistency error was considered to be sufficiently small to result in accurate registrations.

### Limitations

Most of the structures fared well after registration was applied to them in order to represent the motion that occurs during a heartbeat. However, in the evaluated datasets, the caval veins and right atrium of the heart were located very close to the border of the 4D Flow CMR image, and were on a few occasions deformed incorrectly by the registration.

**Fig. 7:**
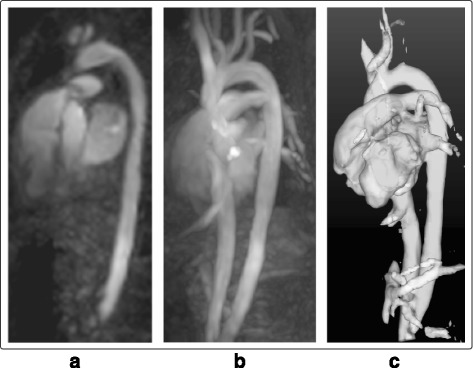
4D PC-MRCA from a dataset acquired without contrast agent: Visualized at a mid-diastolic timeframe. **a** Coronal slice, **b** Maximum Intensity Projection (MIP), and **c** Isosurface rendering

Currently, 4D Flow CMR images are often acquired after injection of a contrast agent for CE-MRA or delayed contrast-enhanced CMR studies. In some cases, even blood pool agents are used [20, 21]. Also in this study, the 4D flow CMR acquisition was preceded by an injection of an extracellular contrast agent for delayed contrast-enhaced CMR. Traces of this contrast agent were still present during the 4D Flow CMR acquisitions, improving the contrast between the blood and the remaining tissues. The presented method was not evaluated in depth on images without any contrast agent. However, preliminary results on this type of datasets showed promising results, and example of such can be seen in Fig. 7. To generate angiographic data from 4D Flow CMR acquired without previous Gadolinium contrast injection, while still maintaining the same quality as the currently presented PC-MRCAs, would most likely require modifications on the technique in order to account for the lower contrast expected in these images.

## Conclusions

The proposed technique to derive a time-resolved three-dimensional phase-contrast Magnetic Resonance CardioAngiography (4D PC-MRCA) outperformed previous techniques to obtain angiographic data from 4D Flow CMR during visual evaluation. The 4D PC-MRCA allows for visualization of the cardiovascular anatomy, including the heart chambers, over the cardiac cycle. This facilitates orientation and enhances visualization of 4D Flow CMR data. Moreover, it might also lead to time-resolved segmentation of this type of acquisitions, which may allow for improved analysis of useful hemodynamic parameters over the complete cardiac cycle.

## Additional files

Additional file 1: 4D PC-MRCA, dataset 1. Isosurface rendering visualization of a 4D PC-MRCA throughout the cardiac cycle. (MP4 745 kb)

Additional file 2: 4D PC-MRCA, dataset 2. Isosurface rendering visualization of a 4D PC-MRCA throughout the cardiac cycle. (MP4 809 kb)

Additional file 3: 4D PC-MRCA MIP at end-diastole. Maximum Intensity Projection (MIP) of a 4D PC-MRCA at end-diastole created using 36 angle projections. (MP4 307 kb)

Additional file 4: 4D PC-MRCA MIP at mid-diastole. Maximum Intensity Projection (MIP) of a 4D PC-MRCA at mid-diastole created using 36 angle projections. (MP4 316 kb)

Additional file 5: 4D PC-MRCA MIP at end-systole. Maximum Intensity Projection (MIP) of a 4D PC-MRCA at end-systole created using 36 angle projections. (MP4 310 kb)

Additional file 6: Isosurface comparison of PC-MRA and PC-MRCA in the heart. Boundaries of the isosurfaces of the PC-MRA and PC-MRCA throughout the cardiac cycle superimposed in a four-chamber view of the 4D Flow CMR magnitude image. (MP4 2264 kb)

Additional file 7: Isosurface comparison of PC-MRA and PC-MRCA in the major vessels. Boundaries of the isosurfaces of the PC-MRA and PC-MRCA throughout the cardiac cycle superimposed in a slice of the 4D Flow CMR magnitude image where the ascending aorta, descending aorta, and pulmonary artery are visible. (MP4 2012 kb)

Additional file 8: 4D PC-MRCA, dataset 3. Isosurface rendering visualization of a 4D PC-MRCA throughout the cardiac cycle for a subject with an enlarged left ventricle and depressed systolic function (Ejection Fraction = 33%). (MP4 1404 kb)

## Abbreviations

CE-MRA: Contrast Enhanced Magnetic Resonance Angiography
CMR: Cardiovascular Magnetic Resonance
MIP: Maximum Intensity Projection
OSI: Oscillating Shear Index
PC-MRA: Phase-Contrast Magnetic Resonance Angiography
PC-MRCA: Phase-Contrast Magnetic Resonance CardioAngiography
WSS: Wall Shear Stress.
